# Illness management and recovery: Clinical outcomes of a randomized clinical trial in community mental health centers

**DOI:** 10.1371/journal.pone.0194027

**Published:** 2018-04-05

**Authors:** Helle Stentoft Dalum, Anna Kristine Waldemar, Lisa Korsbek, Carsten Hjorthøj, John Hagel Mikkelsen, Karin Thomsen, Kristen Kistrup, Mette Olander, Jane Lindschou, Merete Nordentoft, Lene Falgaard Eplov

**Affiliations:** 1 Mental Health Centre Frederiksberg & Mental Health Centre Ballerup, Mental Health Services—Capital Region of Denmark, Copenhagen, Denmark; 2 Competence Centre Recovery and Rehabilitation, Mental Health Centre Ballerup, Mental Health Services—Capital Region of Denmark, Copenhagen, Denmark; 3 Research Unit, Mental Health Centre Copenhagen, Mental Health Services—Capital Region of Denmark, Copenhagen, Denmark; 4 Mental Health Centre Frederiksberg, Mental Health Services—Capital Region of Denmark, Copenhagen, Denmark; 5 Mental Health Centre Hvidovre, Mental Health Services—Capital Region of Denmark, Copenhagen, Denmark; 6 Municipality of Roskilde, Roskilde, Denmark; 7 Copenhagen Trial Unit, Centre for Clinical Intervention Research, Rigshospitalet, Copenhagen University Hospital, Copenhagen, Denmark; Brown University, UNITED STATES

## Abstract

**Objective:**

Illness Management and Recovery (IMR) is a psychosocial intervention with a recovery-oriented approach. The program has been evaluated in different settings; however evidence for the effects of IMR is still deficient. The aim of this trial was to investigate the benefits and harms of the IMR program compared with treatment as usual in Danish patients with schizophrenia or bipolar disorder.

**Method:**

The trial was designed as a randomized, assessor-blinded, multi-center, clinical trial investigating the IMR program compared with usual treatment. 198 people diagnosed with schizophrenia or bipolar disorder participated. The primary outcome was the Global Assessment of Functioning (GAF-F) at the end of intervention and the secondary and explorative outcomes included severity of symptoms and service utilization.

**Results:**

IMR had no significant effect on functioning, symptoms, substance use or service utilization.

**Conclusion:**

This randomized trial contributes to the evidence base of IMR by providing a methodological solid base for its conclusions; however the trial has some important limitations. More research is needed to get a firm answer on the effectiveness of the IMR.

## Introduction

Illness Management and Recovery (IMR) is a curriculum-based rehabilitation program developed to help people with severe mental illness improve their illness self-management and to obtain a decreasing severity of symptoms, increase level of functioning, and achieve prolonged remission [1]. Previously, five randomized trials, investigating IMR have been conducted with varying sizes and design, among others four randomized on an individual level [2–5], whereas one used a cluster randomization design [6].

The findings from four of these trials indicate that IMR is effective in terms of clinical recovery measures. Participants receiving IMR showed significant improvements in psychiatric symptoms, that is positive symptoms, negative symptoms, symptoms of depression [2,4], in coping with symptoms [3], in psychosocial functioning [4], reduced hospital use over time [5], and less suicidal ideation [2] compared with participants who receive treatment as usual. The latest published trial investigating IMR versus an active control group reported that IMR was not significantly different from the control group on symptoms, medication adherence, or service utilization, but there were very low rates of retention in both the IMR and control groups over the study period [6]. The results from these trials should, however, be interpreted with caution. All trials lack one or more desirable aspects of controlled clinical trials, such as power and sample size calculation, outcome assessment by blinded assessors, or systematic approach to handling missing data, resulting in an increased risk of bias. Consequently, the intention with this trial was to add knowledge to the existing evidence of the IMR program by meeting the highest standards of methods in a randomized clinical trial.

The aim of this randomized trial was to investigate the effects of the IMR program compared with treatment as usual in Danish patients with schizophrenia or bipolar disorder. The following hypothesis was tested: Participants in the IMR program would score higher than participants receiving treatment as usual on the Global Assessment of Function scale (GAF-F) at the end of the 9 month intervention period. In this paper, the results on the clinical outcomes: functioning, symptom severity, social functioning, drug/alcohol abuse, and service utilization will be presented. Results regarding self-perceived recovery have been presented elsewhere [7].

## Methods

### Design

The trial was designed as a randomized, assessor-blinded, multi-center, clinical trial of the IMR program compared with treatment as usual in community mental health centers in the Capital Region of Denmark with a post-intervention assessment and a one-year follow-up; see the trial protocol [8].

### Recruitment

Participants were recruited from three community mental health centers (CMHC) in the Capital Region of Denmark. To increase the number of participants enrolled in the study an additional community mental health center was added to the original two. The project was planned in collaboration with the heads of management at the first two of the three CMHC, all of which had shown commitment to implement IMR.

### Participants

Eligible participants were at least 18 years old, receiving services from one of the CMHCs, diagnosed according to the ICD-10 criteria with schizophrenia or bipolar disorder, able to speak and understand Danish, and provided written informed consent. The Present State Examination [9] was used to verify the diagnosis prior entering the trial. Patients were excluded if they had a guardian or a forensic arrangement, or met the criteria for ICD-10 diagnosis of dementia or mental retardation, had an active substance use disorder, lived in a community residential home as treatment as usual is different for this group, or were involved in psychoeducation at the time of inclusion.

### Randomization

After the baseline assessment, participants were randomly allocated to IMR plus treatment as usual or treatment as usual alone. To secure the concealment of the allocation sequence, the randomization was central and telephone-based through an administrative office unrelated to the research team. The allocation sequence was stratified by diagnosis and CMHC. The allocation sequence was computer-generated using permuted blocks varying in sizes of 6, 8 and 10. Randomization was done one at a time and on average the patients in the intervention group waited 87 days (SD 77) before the first group session was held and 62% of the patients waiting under 90 days before the first group session took place.

### Interventions

#### The illness management and recovery program

Patients randomized to the experimental intervention were offered group-based IMR plus treatment as usual (see below). IMR is a curriculum-based program organized in 11 modules which consist of: recovery strategies; practical facts about mental illness; the stress-vulnerability model; building social support; using medication effectively; drug and alcohol use; reducing relapses; healthy lifestyle; coping with stress; coping with problems and symptoms; and getting your needs met in the mental health system. IMR was provided in a group format with weekly one-hour sessions over a period of nine months. Ten patients were assigned to each group which was facilitated by two or three practitioners. IMR was conducted using a closed enrollment group format, such that once the group was started new participants were not enrolled into it (IMR has also been conducted using open group formats). Strategies such as motivational phone calls and in-hospital-sessions were employed to motivate participants to attend and to be engaged in the IMR program. A further description of the IMR program can be found elsewhere [8, 10].

#### Treatment as usual

Participants randomized to the control group received treatment as usual only, which included individually adapted interdisciplinary treatment at the CMHC or in the patient’s own home. Treatment included medication, case management, group therapy (i.e. cognitive behavioral therapy), as well as unstandardized psycho-education. Staff members across the three CMHCs had comparable levels of education and years of experience working with people with severe mental illness. Every patient had a case manager who together with the patient planned the individualized treatment. The patient met with case manager at the CMHC or at home or attended other activities at the community mental health center approximately once a week.

### Outcomes

Baseline assessments were conducted from February 2011 till December 2012 and follow-up assessments from March 2012 till December 2013. The primary outcome was global functioning post-intervention assessed by the Global Assessment of Functioning (GAF-F). The GAF scale can be divided into two scales GAF-F and GAF-S (one that focuses on functioning and one that focuses on symptoms) [11–13]. In this trial the focus of the primary outcome was functioning which is why the GAF-F scale was used. The secondary outcomes were symptom severity on Positive and Negative Syndrome Scale (PANSS) [14] and social functioning on Personal and Social Performance Scale (PSP) [15]. The PANSS has been validated in Danish [14] but not the PSP scale. Exploratory outcomes were symptoms on the GAF-S, depression on the Hamilton Rating Scale for Depression (HAM-6) [16, 17], mania on Young Mania Rating Scale (YMRS) [18], substance abuse (assessed by the case manager), and service utilization based on hospital records.

### Assessment of implementation

The IMR Fidelity Scale [10] was used to assess fidelity to the IMR in all the groups. A multiple data approach was used including interviews, observation of the IMR group, an audit of the patient service records as well as audits of the IMR notes of progress. The fidelity assessments were made half-way through the program (after 4 months) and at the end of the intervention for each IMR group (9 months).

### Power and sample size

Prior to recruitment a sample size of 200 participants was estimated sufficient for detecting a true difference in the IMR and control group of at least 6 points on the GAF-F. The few previous studies using IMR or elements of IMR, where the effectiveness has been assessed by using the total GAF score, have showed a difference from 6 to 10 points [19, 20]. Based on this knowledge we conservatively estimated the true difference in the experimental and control group means to be 6 points on the GAF-F score. The sample size calculation was furthermore based on a power of 80%, an alpha of 5% and a standard deviation (SD) of 15 points [21, 22]. For the secondary outcomes it was determined that the sample size of 200 participants was sufficient to test minimal clinically relevant differences with an alpha of 5% and a power of 80%. See the trial protocol for more information on power and sample size calculation [8].

### Blinding

Post intervention assessments were conducted by assessors who were blind to treatment allocation. Two different assessors performed the assessments, and therefore the assessors rated together to reach agreement before rating patients individually to ensure inter-rater reliability. Analysis of these data showed an acceptable inter-rater reliability (S1 Text). Participants as well as the staff were strongly inculcated not to disclose the allocation status of the participant at the follow up assessment. The statistical analyses were conducted with the two intervention groups coded as A and B, and the Steering Committee drew the conclusions with the blind still intact. In order to secure this blinding the analyses exploring the interactions between degree of participation in IMR and outcomes were carried out after all the other analyses were completed.

### Statistical analysis

The data analysis was based on the intention-to-treat principle. The analysis of difference between the two groups was conducted using analysis of covariance for the continuous primary and secondary outcome measures (GAF-F, GAF-S, PSP, and PANSS). This was estimated as marginal means adjusted for the baseline value of the variable in question [23]. Independent sample t-tests were conducted for the variables concerning substance use, service utilization, that is number and length of hospital admission and number of emergency service visits together with number of visit to the community mental health center (use of treatment as usual) and harm and adverse effect. As service utilization and harms and adverse effects were measured during the study period no baseline mean existed. As missing data is a potential source of bias a strategy of conducting an analysis using multiple imputations were decided beforehand [8]. An analysis of missing data was conducted for the primary and secondary variables GAF-F, PSP, and PANSS and it showed that up to 23% of all observations were incomplete. Therefore, multiple imputation with chained equations under the assumption of data missing at random (MAR) was conducted to enable intention-to-treat analyses. Post-treatment values were imputed for GAF-F, PSP, and PANSS, using baseline values of all three variables, sex, diagnosis, age, community mental health center and intervention group as covariates. The automatic procedure was used and 100 imputations estimated. Per-protocol analysis was performed to see if group attendance influenced the result as proposed in one trial [24]. An analysis using the continuous variable of group attendance for the participants randomized to IMR was made as well as an analysis using a dichotomous variable categorizing attendance into 0–10 sessions or ≥11 sessions. Subgroup analyses tested whether diagnosis or sex interacted with the primary and secondary outcomes. The level of significance for all statistical tests was 0.05. The IBM SPSS Statistics version 19 for Windows was used for statistical analysis and STATA /SE version 13.1 was used for multiple imputations.

### Ethical considerations and information to participants

Patients were recruited from the involved community mental health centers in the Capital Region of Denmark by their case manager supported by a local IMR supervisor. The patients were informed about the on-going trial by a poster in the waiting room and verbally by their case manager. If they were interested in participating they received more information about the IMR program and what their role would be in the controlled trial both verbally and written. Interested participants provided consent verbally and in writing. The case managers were informed about the trial and how to provide information to the patients by the researchers, the local IMR supervisor and their management. The trial was approved by the Ethics Committee in the Capital Region of Denmark (H-1-2010-134) January 20^th^ 2011, before recruiting patients, reported to the Danish Data Protection Agency (RHP-2011-09) during 2011, and registered on www.clinicaltrials.gov (NCT01361698). The authors confirm that all ongoing and related trials for this intervention are registered.

## Results

**Fig 1:**
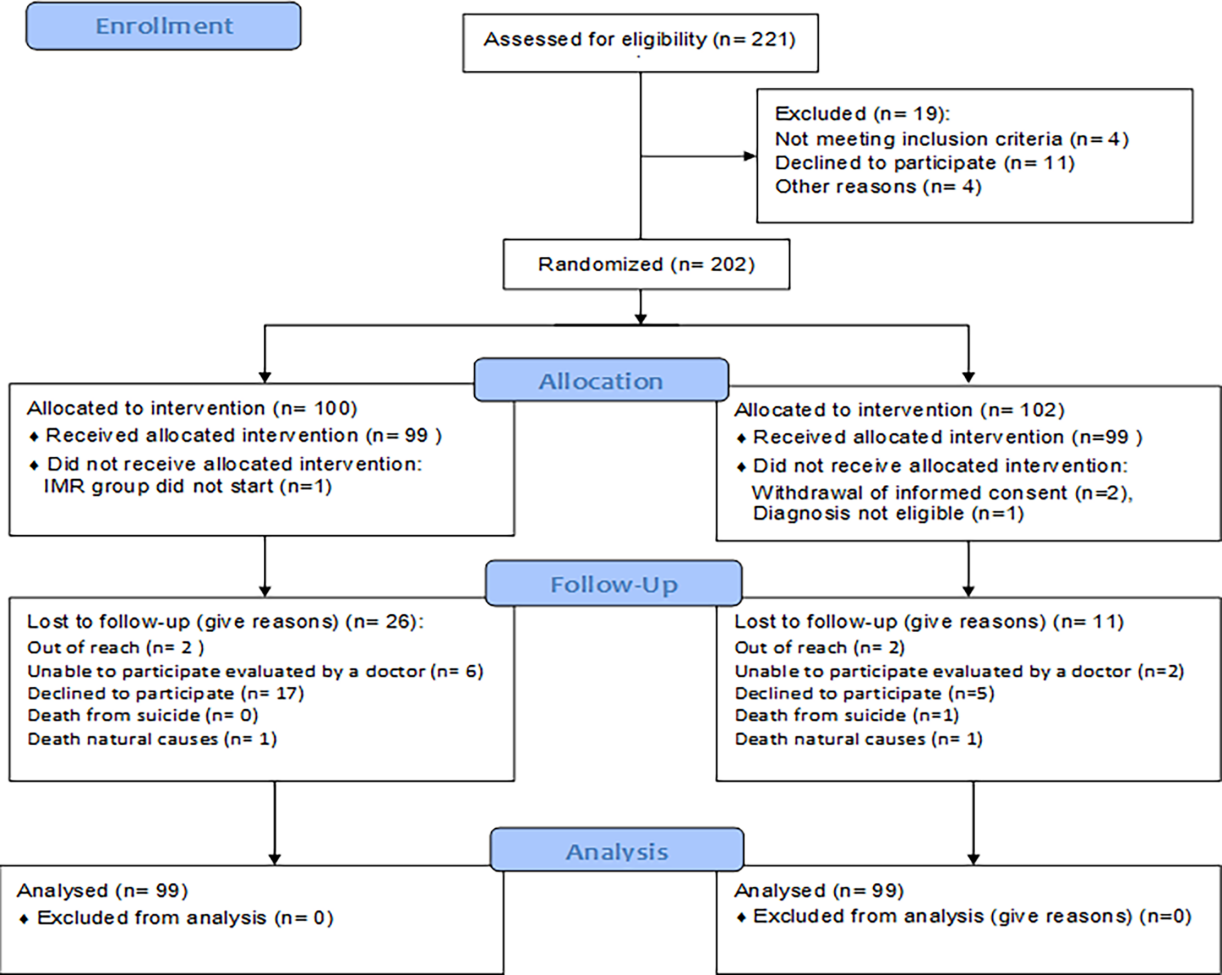
Flow diagram for the Danish illness management and recovery trial.

Fig 1 shows the CONSORT diagram for the participants through the trial. 202 participants were included and randomized but four of them were excluded immediately after randomization: Two participants from the control group withdrew their informed consent, one participant from the control group did not meet the criteria for diagnosis after all, and one participant was assigned as the only participant in an IMR group, that was never conducted and therefore the research group decided to exclude this individual from the trial instead. Therefore, 198 participants entered the trial, 99 participants in each intervention group.

The baseline characteristics of the 198 participants are listed in Table 1, showing an equally distributed in the two groups. A total of 26 participants from the IMR group and 11 from the control group did not participate in the follow-up assessments, a statistically significant difference (χ2 = 7.48, df = 1, p = 0.006). The reason for drop-out was that most patients in both groups simply did not want to continue participating with a higher percentage giving this answer in the intervention group compared to the control group (55% vs. 45%).

**Table 1:** Baseline characteristics of the participants.

	IMR* (N = 99)		TAU^†^ (N = 99)	
Variable	N	%	N	%
**Site**				
CMHC‡ Ballerup	29	29.3	25	25.3
CMHC Gladsaxe	30	30.3	33	33.3
CMHC Frederiksberg	40	40.4	41	41.4
**Sex**				
Female	45	45.5	44	44.4
**Age**				
Age (mean ±SD)	41 (±11.0)		45 (±11.5)	
Age range	20–68		22–77	
**Housing**				
Rented housing	75	75.8	65	65.7
Cooperative dwelling	14	14.1	18	18.2
Owner-occupied housing	8	8.1	10	10.1
Homeless	0	0	0	0
Missing data	2	2	6	6.1
**Employment status**				
Employed	7	7.1	12	12.1
Student	5	5.1	0	0
Unemployed or retired	84	84.8	81	81.8
Missing	3	3	6	6.1
**Education**				
Public School	26	26.3	26	26.3
High school	17	17.2	17	17.2
Vocational training	18	18.2	18	18.2
University	27	27.3	29	29.3
Missing	11	11.1	9	9.1
**Living status**				
Alone	70	70.7	69	69.7
Living with spouse and/or children	19	19.2	26	26.3
Other e.g. co-housing scheme	6	6.1	0	0
Missing	4	4	4	4
**Diagnosis**				
Schizophrenia	76	76.8	75	75.8
Bipolar disorder	23	23.2	24	24.2
**Recent suicide attempt(s)**	2	2	4	4
**Alcohol or drug abuse**				
Alcohol or drug abuse	15	15,2	13	13,1
No abuse	80	80,8	80	80,8
Missing	4	4	5	5.1
**Years since first contact (± SD)**	14 (±10.3)		16 (±10.2)	
Missing	17	17,2	14	14.1

* Illness Management and Recovery

^†^ Treatment as usual

^‡^ Community Mental Health Center

### Exposure to IMR in the intervention group

The exposure to IMR in the intervention group is shown in Table 2. The exposure rate was 57.6%, as 57 of the 99 participants attended 10 or more IMR sessions. Among all the participants mean number of sessions were 16.4 (SD 13.3), compared to a mean of 26.1 (SD 8.1) among the 58, who participated in 10+ sessions. To explore if there were any differences between the non-exposed participants (attending 0–10 sessions) and the exposed participants (attending 10+ sessions) we did some post-hoc analyses to see, if there was a different in the time they had to wait from completion of baseline assessment to beginning the first IMR group as well as differences in baseline characteristics. There were no significant different in the mean waiting-time for the two groups (S1 Table). Furthermore, there were no significant differences in sex, age, housing, employment status, education, living status, diagnoses and substance abuse, but a significant difference in numbers in the two groups when we compared the three CMHCs (S2 Table).

**Table 2:** Exposure to IMR in the intervention group.

Degree of penetration to IMR among the intervention group (N = 99)	n (%)
Participants attending 0 IMR sessions (not engaged)	13 (13.1)
Participants attending only 1 IMR session (minimally engaged)	8 (8.1)
Participants attending 2–9 IMR sessions (engaged but not exposed)	21 (21.2)
Participants attending 10–20 sessions	11 (11.1)
Participants attending more than 20 sessions	46 (46.5)

### Intention-to-treat analyses

Multiple imputations were used to analyze data according to intention-to-treat principle (Table 3). IMR was not significantly different from treatment as usual regarding level of functioning assessed by GAF-F, mean difference: +2.5 points (95% confidence interval (CI): -1.4 points to +6.4points, t = 1.25, df = 1, p = 0.21). For the secondary outcomes, there were no group differences regarding PSP, mean difference: +3.7points (95% CI: -0.8points to +8.1points, t = 1.62, df = 1, p = 0.11), or PANSS, mean difference: -2.7points (95% CI: -8.3points to2.8 points, t = —0.4498, df = 1, p = 0.33).

**Table 3:** Analyses of primary, secondary and explorative outcomes, service utilization, harms and adverse events.

**Intention-to- treat-analyses**											
	IMR*					**TAU**^†^					**P-value**^‡^
	Baseline		Post-intervention		N	Baseline		Post-intervention		N	
	Mean	SD^§^	Mean	SD		Mean	SD	Mean	SD		
*Primary outcome*											
GAF (F) ^||^	40.4	9.3	46.4	14.6	99	40.7	8.3	44.0	13.3	99	0.21
*Secondary outcome*											
PSP^¶^	50.0	11.7	53.3	16.9	99	48.9	11.3	49.6	15.7	99	0.11
PANSS**	63.6	15.0	56.6	20.5	99	64.9	14.3	59.4	19.3	99	0.33
**Complete cases analysis**											
	**IMR**					**TAU**					**P-value**
	Baseline		Post-intervention		N	Baseline		Post-intervention		N	
	Mean	SD	Mean	SD		Mean	SD	Mean	SD		
*Primary outcome*											
GAF (F)	39.9	7.1	45.9	12.3	72	41.0	7.6	44.3	12.8	81	0.21
*Secondary outcome*											
PSP	49.2	11.4	53.0	13.2	71	49.0	10.5	49.5	14.7	79	0.09
PANSS	64.3	15.5	57.1	16.0	69	64.5	13.4	58.3	17.3	76	0.63
*Explorative outcome*											
GAF-S^††^	43.0	9.0	48.2	13.6	63	42.8	9.1	48.2	13.4	75	0.96
HAM-6^§§^	6.9	4.0	5.6	4.5	69	6.3	3.6	5.7	3.9	77	0.50
YMRS^||||^	8.1	5.1	6.9	5.8	69	7.8	5.1	7.2	5.6	76	0.63
	N	%	n	%		N	%	n	%		
Substance use	12	18	9	13	67	10	14	10	14	74	0.28
**Service utilization during the study period**											
			**IMR**					**TAU**			
			Mean	SD	N			Mean	SD	N	
Number of hospital admissions			0.6	1.1	99			0.6	1.7	99	0.92
Length of hospital admissions			13.5	37.1	99			12.5	46.3	99	0.87
Use of emergencies services			1.1	2.6	99			1.0	2.6	99	0.83
Use of treatment as usual			24.1	23.8	93			24.5	20.2	94	0.88
**Harms and adverse events during the study period**											
			**IMR**					**TAU**			
			n	%	N			n	%	N	
Participants attempting suicide			1	1	99			0	0	99	0.28
Participants dying of suicide			0	0	99			1	1	99	0.32
Death (all causes)			1	1	99			2	2	99	0.56

Analyses based on ANCOVA and T-test.

*Illness management and recovery

^†^ Treatment as usual

^‡^ Comparison of means/numbers IMR and TAU group post-intervention

^§^ Standard deviation

^||^ Global Assessment of Functioning- Function subscale

^¶^ Personal and Social Performance

** Positive and Negative Syndrome Scale

^††^ Global Assessment of Functioning–Symptom subscale

^§§^ Hamilton Rating Scale for Depression, 6 items

^||||^ Young Mania Rating Scale

### Complete case analyses

When analyzing data as complete cases, which are all cases where we had observed data on, similar results were found for GAF-F, PSP, and PANSS as in the intention-to-treat analyses, please see Table 2. No differences between the two intervention groups were seen in any of the explorative assessments of GAF-S, HAM-D, and Young Mania Rating Scale.

### Service utilization

Treatment as usual that is the number of visits to CMHC (treatment i.e. meetings with the case manager, meetings with a psychologist or a psychiatrist, or times participating in group activities) was the same for both groups, see Table 3. IMR participants had a mean of 24.1 visits (SD = 23.8) and control group participants had a mean of 24.5 visits (SD = 20.2), respectively. This number covered a wide range where some participants in both groups had no visits and some had more than 130 visits during the follow-up period. Exposure to IMR in the intervention group is shown in Table 2.

Participants in the IMR group had a mean number of days in the psychiatric hospital of 13.5 (SD = 71.1) whereas participants in the control group had a mean number of 12.5 days (SD = 46.3), there was no statistically significant difference. There were no significant differences in the number of times the psychiatric emergency service was used or in number of hospital admissions between the two groups.

### Per-protocol subgroup analysis

The results of the three per-protocol subgroup analyses are listed in Table 4. There was no association between a higher number of sessions attended and the end of intervention GAF-F score (F = 5.7, df = 1, p = 0.49). Subgroup analyses showed no differences in the effect of IMR according to diagnosis or sex regarding the outcomes of GAF-F, PSP or PANSS.

**Table 4:** Subgroup analyses: IMR attendance, diagnosis and sex, end of intervention results.

				**0–10 sessions** **(N = 42)**			**10+ sessions** **(N = 57)**		
Attendance in IMR*			**Mean**	**SD**^†^	**N**	**Mean**	**SD**	**N**	**P-value**
	**GAF-F**		43.8	15.6	20	46.7	10.8	52	0.49
	**PSP**		47.6	18.6	20	55.2	9.9	51	0.11
	**PANSS**		59.7	19.4	18	56.1	14.8	51	0.19
				**IMR**			**TAU**^‡^		
**Diagnosis**			**Mean**	**SD**	**N**	**Mean**	**SD**	**N**	**P-value**
Schizophrenia	**GAF-F**^§^		44.2	11.2	56	42.0	11.5	60	0.11
	**PSP**^||^		50.8	12.6	55	47.5	14.6	59	0.16
	**PANSS**^¶^		60.0	15.7	54	61.1	17.8	57	0.71
Bipolar disorder	**GAF-F**		52.1	14.4	16	51.0	13.9	21	0.79
	**PSP**		60.6	13.0	16	55.5	13.5	20	0.24
	**PANSS**		46.5	12.8	15	49.8	12.7	19	0.57
**Sex**									
Male	**GAF-F**		45.0	11.0	42	41.6	11.6	46	0.10
	**PSP**		55.0	15.0	41	53.0	15.0	45	0.06
	**PANSS**		58.6	16.6	41	61.1	19.0	44	0.43
Female	**GAF-F**		48.1	14.0	30	47.2	13.7	35	0.80
	**PSP**		51.5	11.8	30	47.0	14.0	34	0.59
	**PANSS**		55.0	15.1	28	54.3	13.3	32	0.81

*Illness management and recovery

^†^ Standard deviation

^‡^ Treatment as usual

^§^ Global Assessment of Functioning

^||^ Personal and Social Performance

^¶^ Positive and Negative Syndrome Scale

Analyses based on ANCOVA

### Harms and adverse events

In Table 3 is an overview of harms and adverse effects. There were no significant differences between the two groups in suicide or deaths. Participants in IMR did not differ from participants in the control group in terms of misuse of alcohol or drugs (F = 1.46, df = 1, p = 0.57). Three participants died in the period of follow-up, one of suicide from the control group and two died of natural causes, one from each group. No relation between participating in the IMR trial and the deaths was detected. No further harms or life-threatening conditions were reported during the trial.

### Implementation

Staff specially trained in IMR fidelity assessment from one participating community mental health center was conducting the assessments at the other community mental health centers’ groups and vice versa. The IMR Fidelity mean score across the three participating community mental health centers assessed half-way at 4 months was 4.2 (SD 0.3) indicating good fidelity and the mean score for the end assessment was 4.9 (0.2) indicating high fidelity. A total of 10 IMR groups started and 9 of them completed. One group ended before time because the participants could not come to sessions because of personal reasons (e.g., serious hospitalization, got a job in the day hours, recently had a baby).

## Discussion

This trial investigated the benefits and harms of the IMR program compared with treatment as usual. The trial was designed as an “add-on” study, and therefore patients in the intervention group received IMR as well as “treatment as usual”. Thereby potential difference between the two groups can only be explained by the effect of the IMR program. We found no effect on functioning, symptoms, substance abuse or service utilization. Two of the five earlier studies on IMR also examined the effect of IMR on functioning. One found no effect [5], but the other had a positive result [4]. Furthermore two of the five studies examined the effect on symptoms. Levitt et al. had a conflicting result, as they found a significant effect on a clinicians measured scale, mainly because of a reduction of the level of depression and anxiety, but no effect on a self-report scale [4], whereas Färdig had a positive result [2]. Two studies of the five studies measured IMR effect on substance abuse, and our result is consistent with these earlier results, as they found no effect [4, 6]. Finally, four of the five studies measured the effect on service utilization, three with a result in line with ours, as they found no effect [2, 4, 5], but the last study had a positive result [6]. As the results of all five earlier trials have to be addressed with caution due to a high risk of bias, one might conclude in line with our study that IMR have no effect on functioning, symptoms, substance abuse or service utilization. However the present study also has some limitation, i.e. not as high an exposure to IMR as wanted. Therefore, in order to get a robust answer to whether IMR is an effective program or not, more research is needed, among others there is a need for collecting the evidence from randomized trials of IMR in a systematic review with a meta-analysis, which is under preparation [25].

### Strengths and limitations

This trial has several strengths. The trial was conducted with adequate generation of allocation sequence; adequate allocation concealment; adequate blinding wherever possible; adequate reporting of all relevant outcomes; intention-to-treat analyses; and no for-profit bias [26–28]. The Illness Management and Recovery Fidelity Score indicated that the implementation of the program was satisfactory. A strength of this trial is that prior beginning recruitment a sample size calculation was made. Furthermore, only patients with a validated diagnosis of schizophrenia or bipolar disorder were included in the trial. In the randomization process an external partner conducted the randomization and assured the concealment of sequence and allocation. It is also strength of the trial that most outcome assessments were conducted blinded, so that the knowledge of group status did not influenced the process. The external validity is high because the included participants represent the majority of patients with severe mental illnesses getting treatment in a community.

However, this trial also has some limitations. It is a limitation that 23 percent of the observed data were incomplete because the attrition rate was high. The attrition from research was higher in the IMR group. Our results indicate that the difference in attrition rate mainly is because more participants in the intervention group simply just did not want to participate in the follow-up interview. As 50% of the participants who withdraw from the research in the intervention group had attended zero or only one IMR-group session, one can argue that the main reason for the higher attrition rate in the intervention group is that these patients were not motivated to participate in the study all together not the IMR-intervention or the research interview. To address attrition from research, intention-to-treat analyses were done with multiple imputation. This is the least biased way to deal with missing data [29]. We may also have been too optimistic when planning the present trial as we based the number of participants needed to include on a 6 points difference on the GAF-F scale. Due to this our sample size may be too small, whereby we do not have the power to state if the observed difference of 2.4 is in fact a true difference, but it is most likely not of ‘clinical’ relevance. A study performed after we have started our study, indicate that the minimum clinically important difference for GAF is 4 points [30]. Furthermore, the waiting-time from randomization to the first IMR group session was on average 87 days and this can have affected the results, since it is important to provide psychosocial treatment when a participant is interested and motivated for it, and this may not be the case in the present study as there was a significant delay from the time a participant said yes to the treatment until they started actually started. Therefore we did a post-hoc analysis to see if the ones attending from 0–10 sessions waited longer than the ones attending 10+ sessions had a longer waiting time, but this was not the case, indicating that the waiting time didn’t affect the motivation for participating in the IMR program. Moreover, only 57.6% were fully exposed to the IMR program and this may have influenced the outcome measure. Therefore we did a subgroup analyses to see if the degree of exposure to IMR affected the primary and secondary outcomes. This was not the case. Finally, it is also a limitation that the secondary outcome Personal and Social Performance scale was not validated in Danish. However, the scale has been validated in a number of other countries and seems reliable and valid.

Overall IMR did not differ from treatment as usual in any of the analyzed outcomes. This means that IMR added to treatment as usual in a Danish context does not result in better or worse outcomes for the participants. A possible explanation is that the participants already received a sufficient high quality treatment as part of the usual treatment and perhaps mental health services and the intensity of it in Denmark is somewhat bigger compared to other countries, and that IMR in that context does not add anything further.

Another possible explanation can be found in the theoretic framework for IMR. According to the theory of the IMR program clinical recovery outcomes such as functioning and symptoms remission are considered to be distal outcomes, therefore a longer follow-up period is needed to address the effectiveness of the IMR program properly. Improvements in functioning is supposed to be found after the participant has adapted the new illness management skills into his/hers life. Perhaps the reason why this trial did not find any difference between IMR and the treatment as usual is that the period of follow-up was too brief. Without setting an exact time frame for the IMR theory maybe this trial assessed a distal outcome in a proximal short-time perspective (post treatment). A longer follow-up assessment could perhaps show if IMR is effective according to global assessment of functioning and the other distal clinical recovery outcomes. The trial is therefore also designed to have a longer follow-up and a 12-month after post treatment follow-up is completed currently.

## Conclusion

IMR had no significant effect on the primary outcome GAF-F at the end of intervention (difference of 2.5 points in favor of IMR, P = 0.21). The 95% confidence interval (-1.4 point to +6.4points) makes the possibility of the hypothesized difference of 6 points favoring IMR unlikely. IMR was not significantly different from treatment as usual at end of intervention in terms of clinical outcomes such as level of functioning, symptoms severity, or service utilization.

This randomized trial contributes to the evidence base of IMR by providing a methodological solid base for its conclusions; however the trial has some important limitations. More research is needed to get a firm answer on the effectiveness of the IMR.

## Conflicts of interest

No conflicts of interest that have to be declared.

## Supporting information

S1 Table Waiting time before starting Illness management and recovery among participants in the intervention group. (DOCX)

S2 Table Baseline characteristic of the participants in the intervention group attending up to 10 sessions and 10 and more sessions. (DOCX)

S1 Text Inter-rater reliability. (DOC)

S2 Text The trial protocol. (DOC)

S3 Text CONSORT checklist. (DOCX)
