# Supplementary material for: Illness management and recovery: Clinical outcomes of a randomized clinical trial in community mental health centers
Source: PLoS One. 2018 Apr 5;13(4):e0194027. doi: 10.1371/journal.pone.0194027 (PMC5886399; doi:10.1371/journal.pone.0194027)
Supplement: S1 Table — (DOCX) [file pone.0194027.s001.docx]

**S1 Table. Waiting time before starting Illness Management and Recovery among participants in the intervention group**

|  | **Attendance in IMR*** | | | |  |
| --- | --- | --- | --- | --- | --- |
|  | 0-10 sessions (n = 40) | | 10+ sessions (n = 57) | |  |
| **Waiting time before starting IMR*** | Mean | SD^†^ | Mead | SD^†^ | P-value |
|  | 73.8 | 84.5 | 93.9 | 70.0 | 0.21 |

* Illness Management and Recovery

^†^ Standard deviation
