# Supplementary material for: Illness management and recovery: Clinical outcomes of a randomized clinical trial in community mental health centers
Source: PLoS One. 2018 Apr 5;13(4):e0194027. doi: 10.1371/journal.pone.0194027 (PMC5886399; doi:10.1371/journal.pone.0194027)
Supplement: S2 Table — (DOCX) [file pone.0194027.s002.docx]

**S2 Table. Baseline characteristic of the participants in the intervention group attending up to 10 sessions and 10 and more sessions**

|  | **IMR^*^ 0-10 sessions** | **10+ sessions** | **P-value** |
| --- | --- | --- | --- |
| **Site, N (%)** |  |  | 0.03 |
| CMHC^†^ Ballerup | 17 (40.5) | 12 (21.1) |  |
| CMHC Gladsaxe | 14 (33.3) | 16 (28.1) |  |
| CMHC Frederiksberg | 11 (26.2) | 29 (50.8) |  |
| **Sex, N (%)** |  |  | 0.11 |
| Male | 19 (45.2) | 35 (61.4) |  |
| Female | 23 (54.8) | 22 (38.6) |  |
| **Age, Mean (SD)** | 39.6 (±11) | 42.0 (±11) | 0.42 |
| **Housing, N (%)** |  |  | 0.10 |
| Rented housing | 30 (71.4) | 40 (70.2) |  |
| Cooperative dwelling | 2 (4.8) | 12 (21.1) |  |
| Owner-occupied housing | 4 (9.5) | 4 (7.0) |  |
| Homeless | 0 (0) | 0 (0) |  |
| **Living status, N (%)** |  |  |  |
| Alone | 25 (59.5) | 45 (78.9) |  |
| Living with spouse and/or children | 10 (23.8) | 8 (15.8) |  |
| Other e.g. co-housing scheme | 4 (9.5) | 2 (3.5) |  |
| Missing | 3 (7.1) | 1 (1.8) |  |
| **Employment status, N (%)** |  |  | 0.72 |
| Employed | 3 (7.2) | 2 (2.5) |  |
| Student | 2 (4.8) | 3 (5.3) |  |
| Unemployed or retired | 34 (81.0) | 47 (92.2) |  |
| **Education, N (%)** |  |  | 0.50 |
| Public school | 10 (23.8) | 16 (28.1) |  |
| High school | 9 (21.4) | 8 (14.0) |  |
| Vocational training | 10 (23.8) | 8 (14.0) |  |
| University | 9 (21.4) | 18 (43.9) |  |
| **Diagnosis, N (%)** |  |  | 0.28 |
| Schizophrenia | 30 (71.4) | 46 (80.7) |  |
| Bipolar disorder | 12 (28.6) | 11 (19.3) |  |
| **Alcohol or drug abuse, N (%)** |  |  | 0.34 |
| Alcohol or drug abuse | 8 (19.0) | 7 (12.3) |  |
| No abuse | 32 (76.2) | 48 (87.7) |  |

**^*^**Illness Management and Recovery

^†^ Community Mental Health Center
