# Supplementary material for: Illness management and recovery: Clinical outcomes of a randomized clinical trial in community mental health centers
Source: PLoS One. 2018 Apr 5;13(4):e0194027. doi: 10.1371/journal.pone.0194027 (PMC5886399; doi:10.1371/journal.pone.0194027)
Supplement: S1 Text — (DOC) [file pone.0194027.s003.doc]

Inter-rater reliability

The two raters (HSD and AKWM) have both have rated 12 people. The raters have rated all the objective measurements (GAF-F, GAF-S, PSP, PANSS, YMRS, HAM-6) and have reached an agreement before they have rated patients individually. To judge how well the two raters did agree a Bland-Altman plot for the GAF-F scoring and the PSP scoring was conducted (see below). It seems like the majority of both ratings are in the range of plus/minus two point of the scale which have been considered acceptable in this trial.

**Bland-Altman Plot of agreement of the two IMR-raters on GAF-F and PSP scoring**


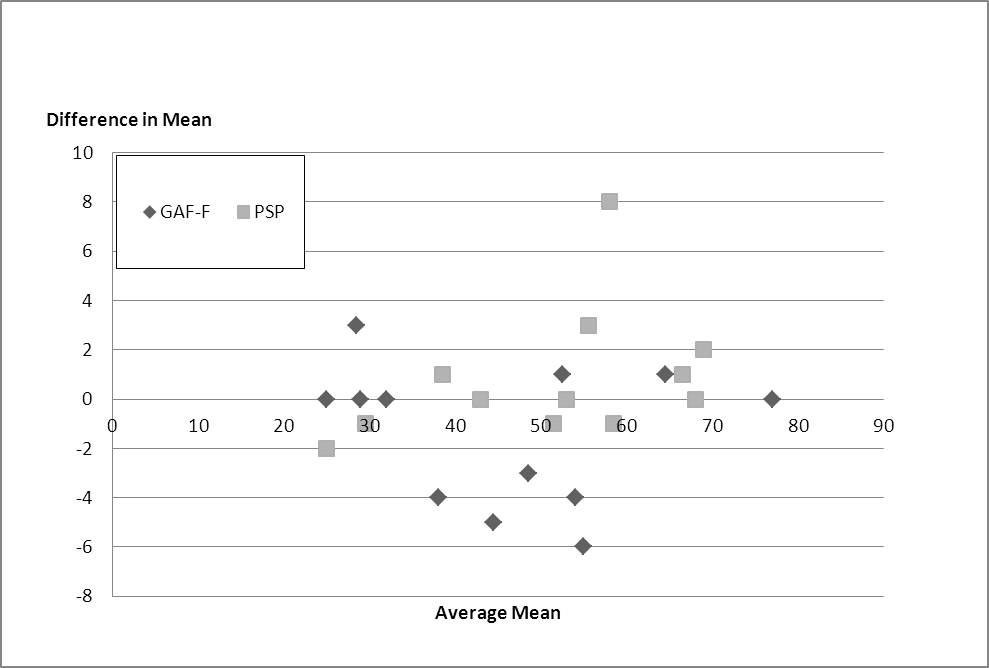
o
