# Supplementary material for: Illness management and recovery: Clinical outcomes of a randomized clinical trial in community mental health centers
Source: PLoS One. 2018 Apr 5;13(4):e0194027. doi: 10.1371/journal.pone.0194027 (PMC5886399; doi:10.1371/journal.pone.0194027)
Supplement: S2 Text — (DOC) [file pone.0194027.s004.doc]

The trial protocol

The trial protocol was written in Danish, but afterward published as a study design article in the open access journal Trials [1], and can be downloaded at <https://trialsjournal.biomedcentral.com/articles/10.1186/1745-6215-12-195>. Below is a translated version of the paragraph “Research design” in the Danish protocol.

Research design

The trial design is carried out as a blinded randomized design involving an intervention group and a control group, see the below figure.

*Overview of the trials of IMR*

Assessed for eligibility for inclusion in the study (n=?) in relation of Inclusion and exclusion criteria

Excluded (n= ?)

Did not meet inclusion

criteria (n=?)

Refused to perticipate (n=?)

Other reasons (n=?)

The number of analyzed (n=?)

Excluded from analysis (n=?)

The reasons to why will be given

Lost to follow-up (n= ?)

Stopped in IMR (n= ? )

**Intervention group: standard treatment + IMR**

Allocated to IMR (n=?)

The number that received IMR (n= ?)

The number that have not received IMR(n= ?)

The reasons to why will be given

**Allocation**

**Analyses**

**Follow-Up**

**9 and 21 mdr. after baseline**

**Enrollment –** the patient gives informed consent

Randomization (n=200)

**Control group: standard treatment**

Allocated to control group (n=?)

The number that received standard

treament in the control group (n= ?)

The number that have not received standard

treatment in the control group (n= ?)

Lost to follow-up (n=?)

Stopped standard treatment (n=?)

The number of analyzed (n=?)

Excluded from analysis (n=?)

The reasons to why will be given

**Baseline Measurements**

In order to ensure the internal and external validity, the study design is based on CONSORT criteria (CONsolidated Standards Of Reporting Trials) of non-pharmaceutical trials [2]. Both the control group and the intervention group will be offered the standard treatment. The participants allocated to IMR will be offered IMR additionally.

### Participants

Participating patients:

The following criteria for inclusion are chosen to maximize the possibility of generalization the results in comparison to the general population of people diagnosed with schizophrenia and bipolar disorder.

Inclusion Criteria:

1. Sex: Adults of both genders
2. Age: Participants must be at least 18 years of age, there is no upper age limit
3. Psychiatric treatment: Associated with one of the two participating community mental health centres, Community Mental Health Center Frederiksberg-Vanløse or Community Mental Health Center Ballerup-Egedal-Herlev
4. Diagnose: Diagnosed following the ICD-10 criteria of schizophrenia (F.20) or bipolar disorder (F.31)[[1]](#footnote-2).
5. Language: Able to speak and understand Danish
6. Informed consent: To give informed consent verbally and in writing.

**Exclusion criteria:**

1. Guardian: Patients will be excluded if they have a guardian or a forensic psychiatric arrangement
2. Massive abuse: The Person has within the past months showed up visible affected by alcohol/drugs for two meetings with practitioner or primary care provider. The exclusion criteria applies to those with a large-scale substance abuse of drugs and/or alcohol that leads to problems for himself, relatives, other patients and staff members. Patients will only be excluded from the trial for as long as they have an ongoing abuse. First, they will be offered treatment for their abuse and then they may be included in the trial subsequently if they met the given criteria.
3. Other diagnoses: Dementia (F.00-F.03), mental retardation (F.70-F.79)
4. Home: The person lives in supported housing. Patients are only excluded from the trial while they live in supported housing, because the treatment here is a different one from the standard treatment in Community Mental Health centers.
5. Other treatment: The person is in an ongoing psycho-educational course (DPC Frederiksberg-Vanløse) or group offer for patients with bipolar disorder (DPC Ballerup-Egedal-Herlev).

This exclusion criterion is chosen because of the overlap between the teaching in IMR and the two abovementioned group offers. However, patients can be included in the trial if they have previously been in the two groups/when they have completed the two group offers.

1. Forensic patients: The person has judicial precaution regarding the psychiatric treatment and can therefore not participate in a research project based on voluntary participation.
2. Not given informed consent.

Visitation:
Patients will be recruited from four mental health centres in the Capital Region of Denmark: Distriktspsykiatrisk Center Ballerup-Egedal-Herlev, Distriktspsykiatrisk Center Gentofte, Distriktspsykiatrisk Center Gladsaxe-Værløse and Distriktspsykiatrisk Center Frederiksberg-Vanløse. The clinicians in the four mental health centers calls in the patient, assesses the patient respectively in relation to inclusion- and exclusions criteria and the patient will be informed about the participation in the trial. Each potential participant is interviewed with the diagnostic tool Present State Examination [3] (PSE) to evaluate whether the patients diagnose meets the inclusion criteria. The clinical interview is conducted by a psychiatrist alternatively a nurse or psychologist with minimum five years of clinical experience in psychiatry. Nurses and psychologists will receive training in interviewing with PSE. The PSE interview will take approximately 1 hour.

Based on that it is determined whether the patient can be included. After thorough information and consideration the participating patients has to sign an informed consent, see a more detailed description in chapter 6. Then baseline data is obtained for the outcome of HSD, etc., See section 2.5. prior randomization, see section 2.8. Participants in the trial will receive no remuneration for participation.

### Intervention

IMR-group:

Patients randomized to the IMR group are besides the IMR-course also offered the standard treatment in a community mental health centre.

From January 2011 - December 2013 IMR will be implemented in the two participating community mental health centre. The aim of IMR is to:

- Inspire hope that change is possible
- Develop a relation with the care provider team based on cooperation
- Help the participants to set personal and meaningful measure, they can strive to achieve
- Communicate information about mental illness and treatment options
- Develop skills to prevent relapse, manage stress and master symptoms
- To inform about where you can get the necessary support
- To help people develop or strengthen their natural resources, when it comes to managing their disease and pursue their goals

IMR looks at the participant’s individual opportunities and ability to set personal goals and pursue them as an essential part of recover. The programs first model of education ”Strategies for recovery” is therefore about helping the participants to define what recovery means to them and to cover what their goals and what dreams are important to them. During the entire educational course, therapists must contribute by helping the participants to set individual meaningful goals and follow up on those as part of transforming the action-enhancing vision of recovery.

Concrete the IMR is a variety of strategies geared towards better cooperation between the individual and the professional with the aim to strengthen the individual’s ability to handle the disease, reduce the number and difficulty of relapse, and pursue their personal goals. The program extends over 9 months with weekly sessions in group of 10 people, and with the involvement of the relatives, if the participant finds it relevant. IMR consists of 11 modules where of number 11 is a later added module that has not yet been tested as much as the others, see the below table .

*Modules in Illness Management and Recovery*

| Module | Number of lessons |
| --- | --- |
| Strategies for recovery | 2-4 |
| knowledge about mental illness | 2-4 |
| The stress-vulnerability model and treatment strategies | 2-4 |
| To build social support | 2-4 |
| Effective use of medicine | 2-4 |
| Use of drugs and alcohol | 1-6 |
| To reduce relapse | 2-4 |
| To master stress and common issues | 2-4 |
| Mastering symptoms | 2-4 |
| To cover ones needs met in the mental health | 2-4 |
| Healthy lifestyle | 2-4 |

The program will be implemented in a combination of educational, motivational, and cognitive-behavioral teaching strategies and homework assignments developed collaboratively with the participant. All of the modules are approximately built the same so that the entire teaching process follows a predictable pattern. A workbook is made with detailed instructions for the teaching and furthermore teaching material in the form of hand-outs for each training module. In regard to the project the workbook is translated into Danish.

Participating staff (those in the intervention):

A total of 24 staff members trained from the two Community Mental Health Centre will be trained in the use of IMR distribution as follows: 16 staff members (super users), 4 from the two mental health managements and 2-4 leaders in staff. The 16 staff members, hereinafter referred to as super users, will be responsible for the education of the patients, and teach other staffs in IMR with the acquired knowledge they then have later on. The super uses will be picked out by the two managements from the community mental health centres with the following criteria: Strong English skills (the staffs training will be in English), good teachers, motivated, stubbornness, big anchor in center, as well as knowledge of cognitive therapy.

The staff is taught in the autumn of 2010 and 2011 with two days of training by Harry Cunnigham from Mental Health Center of Greater Manchester, New Hampshire, USA. The teaching of the staff will take place before the project begins. The training will be followed up on with two days of training after 6 months after the first IMR group has started and possibly again after 12 months. In addition to this the staff will receive tele supervision from Harry Cunnigham or from the staff associated with the Mental Health Center of Greater Manchester for a year of. In the beginning supervision will be every 1-2 weeks and then less frequently, expectedly one time per month upon the end. Tele Supervision takes place at each community mental health center. Two hours of supervision is set off each time, thus it is possible to have both a pre- and post-talk to counter problems related to the language. Furthermore, a network group will be established with the participation of all the super users from PC Ballerup and PC Frederiksberg. The goal is to support the implementation of IMR and for the super users to get to know each other across the two centers. From each community mental health center a coordinator is pointed out, on which they have special responsibility in relation to the practical implementation of IMR locally and will facilitate the network meetings.

Control group (standard treatment):

Standard treatment consists of individually tailored, interdisciplinary treatment which the community mental health centres has offered for several years. The standard treatment in the community mental health centres auspices are medicine, individual supportive conversations with patients or district psychiatry facilities, individual occupational therapy as well as training and conversation groups, see Appendix A1 for a detailed description of the included community mental health centres and the treatment.

### Outcome measures

The effect of IMR compared with treatment as usual is measured on both primary and secondary outcome. Measurements are made at baseline and after 9 months by a blinded researcher (HSD), which will be thoroughly trained in all outcomes.

The primary outcome is GAF-F with the time 9 months (Intervention completion), while the secondary outcome are functions, symptoms and personal recovery with the time 9 months and the possibility of a one-year follow-up (21 months after randomization). Personal recovery is a multidimensional concept, why the involvement of proxy variable as for example hopes and empowerment would be relevant to measure its different aspects [4]. The aim is to measure function and symptoms with the researcher's assessment by interviewing the patient, while the goals of personal recovery are obtained from the participant's self-assessment [5]. The interview will last max. 2 hours, and the questionnaires which the patient must fill out himself will maximum take an hour to complete. The patient fills out self-assessment at home and the blinded researcher (HSD) will then with the patient review the completed forms to identify any comprehension.

Primary outcome

Level of function

- Global Assessment of Functioning (GAF) [6], The GAF scale can be divided into two scales GAF-F and GAF-S (one that focuses on functioning and one that focuses on symptoms). GAF-F [7] is the primary outcome. GAF-F is an expression of clinical recovery measured by functional level, and completed by HSD after an interview with the patient.

Secondary outcomes

Functioning

- Personal and Social Performance (PSP) [8]. PSP measuring social functioning compared to four domains: *socially useful activities*, *personal and social relationships*, *self-care* and *disturbing and aggressive behavior*. PSP completed by HSD after an interview with the patient.

Symptom

- Positive and Negative Symptom Scale (PANSS) [9]. This scale aassesses the level of a severity of positive and negative symptoms, and completed by HSD after interview with the patient.
- Hamiltons Depressionsskala (HAM-D_6_) [10]. This scale assesses the level of symptoms of depression and completed by HSD after interview with the patient.
- Youngs Maniskala. This scale assesses severity of manic episodes, and completed by HSD after interview with the patient [11].
- Alcohol / drug intake - registers of primary therapist

Personal recovery

- lllness Management and Recovery Scale. This scale is especially developed to the IMR program and assesses on all relevant areas including on personal goals, knowledge about the disease, hospitalizations, use of alcohol or drugs, function, symptoms, stress and coping [12]. Measured by patient self-assessment and primary care professional's assessment.
- Mental Health Recovery Measure [13]. This scale assesses is a measurement of recovery in a general level and not specific attached to the IMR program, by involving different aspects related to personal recovery. Measured by patient’s self-assessment.
- Adult State Hope Scale [14]. This scale is a self-report scale which measures hope and optimism by patient’s self-assessment.

Security measures

To assess how safe the intervention is the below variable is registered:

- Suicide - registered by the case manager.
- Suicide - registered via the green system.
- Life-threatening conditions (other reasons than suicide attempt) - recorded by the case manager.
- Death (all causes - not only suicides) - registers via the green system.

Service utilization

Through all three studies the participants will be followed by hospital records ("The Green System"), on which there are yearly measurements on:

- Number of admissions
- Inpatient days
- Use of outpatient services

Other registration

Satisfaction

- Clients Satisfaction Questionaire [15]. This scale assesses the participants’ satisfaction with the psychiatric services and is measures from patient’s self-assessment.

Program fidelity

- Fidelity in relation to the concept of IMR the teacher is ensured by the use of llness Management Fidelity Scale [16]. To ensure that the staff in standard treatment is not using tools from IMR Illness Management Fidelity Scale is also used in relation.
- Specifically, this measurement will be made after completion of the IMR-process of the conductor from the second community mental heath centre, as the coordinator for Frederiksberg-Vanløse assess Ballerup Herlev-Egedal and vise versa.

**Covariates**

Clinical features

- Diagnose
- First contact with the psychiatric treatment system
- Attempted suicide

Social features

- Housing
- Cohabitation
- Education and support

**Case Record Form**

For all the participants there will be a *case record form*. Here, all data about the participant is stored i.e. outcome, harmful effects and covariates. Also the use of standard treatment for both groups will be register here, see Appendix A2 as well as the IMR-teaching attendance will be register for the intervention group.

Follow-up

It is planned to contact the participants in the attempt to do a follow-up nine months after baseline and 21 months after baseline i.e. one year after the intervention is completed. Furthermore, follow-up for 10 years with a naturalistic design, i.e. participants in the control group will be offered IMR in the follow-up period.

### Side effects, risks, cons, drop-out

Previous experience with IMR does not suggest that this rehabilitation program is associated with side effects for patients. It is estimated that by participating in this study it may be a smaller burden for the patient to answer questionnaires and participate in interviews. To achieve minimum risk of inconvenience for the participants, the various outcomes are implemented flexibly with the possibility of taking a break or split the interview / filling out questionnaires over two days. If the participants find the interview stressful or uncomfortable, they can simply step out of the trial, without it affecting their possibility to receive future treatment, see below. The participants in the IMR Group, who have social difficulties may find participation in the IMR Group overwhelming or difficult, this is something that will be work through and on at the IMR-course.

Drop-out
If the enrolled participant for some reason does not want to participate, there are three options for drop-out from the trial:

1. The participant does not wish to participate in the IMR or standard treatment, but would like to participate in the trial and follow-up outcome after 9 months and 21 months. Baseline and follow-up data on participant will be included in the analysis.
2. The participant does not wish to participate in the IMR or standard treatment, but would like to participate in the trial, but the person does not want to participate in follow-up after 9 months and 21 months. Baseline data regarding the participant will be included in the analysis.
3. The participant does not wish to participate in the IMR or standard therapy, and will no longer participate in the trial. All data regarding the participant is deleted and will not be included in the analysis.

### Strength calculation and study population

Based on administrative records the two mental health centers serves in total 1050 patients with a patient movement at least 350 patients per. year. The calculation of the study is based on Power and Sample Size Program. The continuous outcomes GAF-F is the primary variable. The trial will be designed so that there is one control patient in the intervention. The strength is set to 0.8, and the significance level to 5%. In a previous randomized trial on psychoeducation in Denmark, where GAF-F was the primary goal, a standard deviation of 15 appeared, which we here are using as a guideline. The effect of the few trails that contains IMR or parts of IMR intervention shows an effect of 6-10 on GAF [17]. Therefore we estimate a slight conservative effect of this intervention on 6, i.e. it’s estimated as the true difference between the average of the experimental and control groups after the intervention. With these assumptions, the study is estimated to having to count 200 people. There are made similar strength calculations for the following outcomes: PSP, PANSS, Hamiltons Depressionsskala, Illness Management and Recovery Scale and Mental Health Recovery Measure and they showed that the included number of 200 individuals is of sufficient strength when showing an effect of these objectives. For other outcomes strength calculation is not made, since they are considered clinically relevant and therefore should be included in the trial.

### Randomization

Randomization in the intervention group and the control group is done via a central computer-based randomizing where the local coordinator or a secretary calls Copenhagen Trial Unit and provides factual information about the patient. In Copenhagen Trial Unit (where the trial is registered as IMR-DP-228) this information is entered in a computer system and then assigned to the patient via the computer system either IMR or standard treatment.

The participants stratified by diagnosis and center so that there are equally numbers in the control group and intervention group under each disease category and from each center. The mental health centers are divided accordingly to which mental health center they belong to: that means DPC Gladsaxe-Værløse, Ballerup-Egedal-Herlev and Gentofte belongs under PC Ballerup, and DPC Frederiksberg-Vanløse belongs under PC Frederiksberg. This gives four strata:

1) Center 1, diagnose A
2) Center 1, diagnose B
3) Center 2, diagnose A
4) Center 2, diagnose B

### Blinding

The staffs in the mental health center are after randomization not blinded from if the patients have received the intervention, and which patients has not. By contrast, the research group will be blinded to which group the patients are in under the evaluative interviews. It will be enjoined that the staff and patients do not talk about the patients' treatment and relation to the research project by the evaluative interviews but only relate to the issues in relation to efficacy measures. HSD will after the interview guess whether the patient is an IMR participant, and this will be registered and compared with the factual status. All data will be entered by an academic employee who is not a part of the research team, and in multiple sets of data, so that researchers do not have access to the data from the IMR-participation in the analysis of affect. At the statistical analysis the researchers will be blinded, such that the two groups will be coded with an "X" and an "Y.” The code for what is IMR and what is control group will be blinded to the researchers throughout the analysis phase and under writing of the conclusion.

### Statistical analysis

Data analysis will be based on intention-to-treat principle, i.e. Data from all patients will be included in the care provider group with possibly multiple imputations, which patients are randomly assigned to. A non-response analysis will be carried out. This analysis estimates the distribution of missing data based on of the information gathered in previous interviews and by utilizing information on outcome in patients from the database who shared the same characteristics with those who did not participate in follow-up. The condition for using this model is that the drop-out is randomly distributed among those who share the same baseline characteristics. Baseline values ​​of variables are automatically included in the model. The continuous outcomes will be analyzed with repeated measurements model with unstructured variance matrix. The differences between the two groups will be examined using the Student t-test, Mann-Whitney U test and Chi-Square and the variances tested using ANOVA. Dichotomous outcomes will be analyzed using logistic regression analysis with baseline values ​​of variables included as covariates [18, 19].

# References

1. Dalum HS, Korsbek L, Hagel Mikkelsen J, Thomsen K, Kistrup K, Olander M et al. Illness management and recovery (IMR) in Danish community mental health centres. [Trials.](https://www.ncbi.nlm.nih.gov/pubmed/?term=Illness+management+and+recovery+(IMR)+in+Danish+community+mental+health+centres.+Trials.) 2011 Aug 17;12:195. doi: 10.1186/1745-6215-12-195.
2. Boutron I, Moher D, Altman DG, Schulz KF, Ravaud P. Methods and Processes of the CONSORT Group: Example of an Extension for Trials Assesing Nonpharmacologic Treatments. Annals of Internal Medicine 2008; 148(4): 60-66.
3. The SCAN Advisory Group. Present State Examination, Short Version for Clinical Use. Based on SCAN (Schedules for Clinical Assessment in Neuropsychiatry, Version 2, American Psychiatric Press, Inc. 1994.
4. Johnson DL. A Compendium of Psychosocial Measures. New York: Springer Publishing Company: 2010.
5. Elsass P, Ivanouw J, Mortensen EL, Poulsen S. & Rosenbaum B (red.). Assessmentmetoder – Håndbog for psykologer og psykiatere. København: Dansk Psykologisk Forlag: 2006.
6. Diagnostic and statistical manual of mental disorders, 4th edition. Washington DC: American Psychiatric Association, 1994.
7. Karterud S, Pedersen G, Loevdahl H, Friss S. Global Assessment of Functioning – split version (S-GAF) background and scoring manual. Olso: Department of Psychiatry, Ullevaal University Hospital, 1998.
8. Nasralla H, Morosin P & Gagnon DD. Reliability, validity and ability to detect change of the personal and Social Performance scale in patiens with stable schizophrenia. Psychiatry Research 2008; 161:213-224.
9. Kay SR, Abraham, Fiszbein A, Qpler La. The Positive and Negative Syndrome Scale (PANSS) for Schizophrenia. Schizophr Bull 1987;13 :261-76.
10. Bech P, Licht R W, Stage KB. Rating Scales for affektive lidelser. Hillerød: Psykiatrisk Forskningsenhed, Psykiatrisk Sygehus Hillerød, 2005
11. Young RC, Biggs JT, Ziegler VE, Meyer DA. A rating scale for mania: Reliability, validity and sensitivity. Br J Psychiatry. 1978;133:429–435.
12. Salyers MP, Godfrey JL, Mueser KT & Labrioala S. Measuring Illness Management Outcomes: A psychometric Study of Clinician and Consumer Rating Scales for Illness Self Management and Recovery. Community Mental Health Journal 2007; 43 (5): 459-480.
13. Young SL, Ensing DS & Bullock WA. Mental Health Recovery Measure. In: Ralph RO, Kidder K & Phillips D (eds.) Can we measure recovery? A compendium of recovery-related instruments Cambridge MA: The evaluation center @ HSRI: 2000.
14. Snyder CR, Sympson SC, Ybasco FC, Borders TE, Babyak MA & Higgins RL. Development and Validation of the State Hope Scale Journal of Personality and Social Psychology 1996, Vol. 70, No. 2, 321-335.
15. Larsen DH, Attkison CC, Hargreaves WA et al. Assessment of client/patient satisfaction: development of a general scale. Eval Progress Plan 1979;2:197-207.
16. Gingerich S, Mueser KT. Illness management and recovery. In: Drake RE, Merrens MR, Lynde DW, editors. Evidence-Based Mental Health Practice: A Textbook. New York, NY: Norton; 2005. pp. 395–424.
17. Haddock G, Barrowclough C, Tarrier N, Moring J, O’Brien R, Schofield N, Quinn J, Palmer S, Davies L, Lowens I, McGovern J & Lewis S. Cognitive-behavioural therapy and motivational intervention for schizophrenia and substance misuse. 18-month outcomes of a randomised controlled trial.British Journal of Psychiatry 2003; 183: 418-426.
18. Rothmann KJ. Epidemiology - An Introduction. Oxford: Oxford University Press: 2002.
19. Kreiner S. Statistisk Problemløsning. København: Jurist- og Økonomiforbundets Forlag:1999.

1. The IMR program is designed for people with schizophrenia, bipolar disorder, schizoaffective disorder and severe depression. We have chosen not to include individuals with schizoaffective disorder and severe depression in this study, since there are not enough people in the two districts to create statistical power by stratification. [↑](#footnote-ref-2)
